# Phylogenetic Reconstructions Based on Mitogenomes Reveal the Paraphyly of the Subfamily Isotominae of Isotomidae (Collembola: Entomobryomorpha)

**DOI:** 10.3390/genes17020166

**Published:** 2026-01-30

**Authors:** Yuhang Cheng, Chunyu Zhang, Donghui Wu, Zhijing Xie, Bing Zhang

**Affiliations:** 1 Key Laboratory of Wetland Ecology and Environment, State Key Laboratory of Black Soils Conservation and Utilization, Northeast Institute of Geography and Agroecology, Chinese Academy of Sciences, Changchun 130102, China; 2 University of Chinese Academy of Sciences, Beijing 100049, China; 3 School of Grassland Science, Beijing Forestry University, Beijing 100083, China; 4 Key Laboratory of Vegetation Ecology, Ministry of Education, Northeast Normal University, Changchun 130024, China; 5 State Environmental Protection Key Laboratory of Wetland Ecology and Vegetation Restoration, School of Environment, Northeast Normal University, Changchun 130024, China

**Keywords:** homoplasy, lineage delimitation, soil invertebrates, soil fauna, springtails, taxonomy

## Abstract

Background: Isotomidae is one of the most common Collembola families, comprising 1484 species belonging to four subfamilies: Isotominae, Proisotominae, Anurophorinae, and Pachyotominae, while the subfamilial classification remains contentious and lack of molecular phylogenetic evidence. Methods: We sequenced and assembled the mitochondrial genomes (mitogenomes) of three species (*Parisotoma* sp., *Folsomia* sp. 1, and *Folsomia* sp. 2. Combining these with 10 mitogenomes available from GenBank, we reconstructed the phylogeny of Isotomidae based on a dataset of 13 species representing all four subfamilies. Results: These new mitogenomes, with lengths of 15,741 bp, 16,295 bp, and 16,765 bp, respectively, exhibit the typical metazoan gene set (13 PCGs, 22 tRNAs, 2 rRNAs) and show high structural conservation with other Collembola species. However, phylogenetic analyses based on concatenated protein-coding genes revealed significant incongruence with traditional classification. While Isotomidae was recovered as monophyletic, both Isotominae and Anurophorinae were recovered as paraphyletic. Specifically, *Parisotoma* sp. formed a distinct lineage closer to the derived subfamilies than to the core Isotominae, and the representative of Pachyotominae (*Paranurophorus simplex*) was recovered nested within Anurophorinae, suggesting potential subfamilial misclassification or paraphyly. Furthermore, *Proisotoma minuta* was identified as an independent sister lineage to the Anurophorinae + Pachyotominae clade. Conclusions: Our findings suggest that the current subfamily boundaries are not natural and that key diagnostic traits, such as furcal structure, likely reflect symplesiomorphies or various forms of homoplasy-including convergent evolution, parallelism, and evolutionary reversals—rather than unique synapomorphies defining monophyletic groups. This study provides essential genomic resources and highlights the need for an integrative taxonomic revision of Isotomidae that incorporates both molecular and morphological data, with particular emphasis on redefining subfamilies boundaries and reassessing diagnostic morphological traits.

## 1. Introduction

Collembola (springtails) are a widely distributed group of arthropods, with over 9000 described species classified into 33 families and 4 orders [1]. The systematics of Collembola have traditionally been based on comparative morphology [2,3]. However, challenges such as uncertain homology, intraspecific variation and structures that are difficult to observe have limited the reliability of morphological characters for species diagnoses and phylogenetic inference [4,5,6]. Consequently, many researchers have proposed the use of multi-gene molecular markers to improve the accuracy and reliability of species identification [6,7,8]. The metazoan mitochondrial DNA (mtDNA) is typically a circular molecule, serving as the primary genetic material located outside the nucleus but within the mitochondria [9]. With the advancement of high-throughput sequencing technology, the mitochondrial genome (mitogenome) has become an essential tool for studying the evolution, phylogeny, and biogeography of springtails [10,11]. Recent studies have increasingly employed mitogenomic data to resolve deep phylogenetic relationships within Collembola, yet sampling remains sparse for many families [12,13]. Nevertheless, only a few dozen complete mitogenome sequences of Collembola are currently available, covering a limited number of species. This scarcity hinders comprehensive research into biodiversity, phylogeny, and species evolution. This scarcity hinders comprehensive research into biodiversity, phylogeny, and species evolution, underscoring the critical importance of constructing both mitochondrial and nuclear genome database.

Representatives of the family Isotomidae (Schäffer, 1896) are among the most common Collembola families in the Palearctic region and are often dominant in both natural and anthropogenically disturbed soil habitats across temperate and subtropical Asia [14]. This family comprises 1484 species worldwide (as of 4 December 2025) and includes four subfamilies: Isotominae (Schäffer, 1896), Proisotominae (Stach, 1947), Anurophorinae (Börner, 1901), and Pachyotominae (Potapov, 2001) [1]. To date, only ten complete mitochondrial genomes are available in the public GenBank database. A recent study by Xie et al. [15], using molecular markers, highlighted phylogenetic inadequacies in this traditional subfamily classification and pointed out that limited taxon sampling hinders robust conclusions. To directly test these proposed phylogenetic hypotheses and expand the genomic resources for this family, we newly sequenced the mitogenomes of three Isotomidae species. Combined with previously published mitogenomic data of representatives from the same family, we conducted comparative mitogenomic analyses and reconstructed a phylogeny with an expanded taxon sampling. The specific objectives of this study are to expand the mitochondrial genome database for Isotomidae and to reconstruct a more comprehensive phylogeny using both Maximum Likelihood and Bayesian Inference across multiple datasets (i.e., PCG and PCG + RNA matrices), in order to evaluate the phylogenetic relationships of the traditionally recognized subfamilies.

## 2. Materials and Methods

### 2.1. Sample Collection and Identification

Specimens for this study were collected in July 2023 from the Saihanba Mechanical Forest Farm in Chengde City, Hebei Province, China. Soil samples from different forest types were obtained using a soil auger with a diameter of 7.5 cm. Soil-dwelling collembolans were extracted using the Tullgren funnel method (40 W incandescent bulb, 24 h, 20 mesh sieve), and preserved in 95% ethanol. Approximately 100 specimens were collected. Voucher specimens (Accession No. SHB-2023-FS01 to SHB-2023-FS03) were deposited in the Insect Collection of the Northeast Institute of Geography and Agroecology, Chinese Academy of Sciences. Morphological identification was conducted based on the taxonomic system of Deharveng [16] and the online resource (http://collembola.org/, accessed on 1 December 2026). However, due to the high morphological conservatism of these genera and the presence of potential cryptic diversity in the study region, species-level identification was not definitively assigned to avoid taxonomic misinterpretation. Instead, they are treated as distinct molecular operational taxonomic units supported by both mitochondrial genome and divergence in the mitochondrial cytochrome c oxidase subunit I (*COI*) gene. To confirm morphological assignments and ensure the genetic homogeneity of pooled samples for sequencing, a preliminary molecular identification was performed using *COI* gene.

### 2.2. DNA Extraction

To ensure taxonomic accuracy and genetic homogeneity, genomic DNA was initially extracted from each individual separately using an Animal Tissue Genomic DNA Extraction Kit (Jingze Biotech, Beijing, China). The concentration and purity of the extracted DNA were assessed using a NanoDrop 2000 spectrophotometer (Thermo Fisher Scientific, Waltham, MA, USA) and Qubit 4.0 Fluorometer (Thermo Fisher Scientific, USA). Samples with an A260/280 ratio between 1.8 and 2.0 and a total DNA amount >1.0 µg were used for downstream analysis. Initially, the *COI* gene was amplified using the universal primers LCO1490/HCO2198 under the following PCR conditions [17]: pre-denaturation at 96 °C for 3 min; 35 cycles of denaturation at 95 °C for 10 s, annealing at 46 °C for 30 s, and elongation at 72 °C for 1.5 min, and a final extension at 72 °C for 5 min. Then, the PCR products were maintained at 4 °C. Amplification products were verified via 1% agarose gel electrophoresis, purified, and subjected to Sanger sequencing by Ruibo Xingke Biotechnology Co., Ltd. (Beijing, China). Individuals confirmed as morphologically consistent and possessing 100% *COI* sequence identity (Table S5) were selected, and their *COI* sequences were used as seeds for subsequent assembly. For Illumina sequencing, DNA from 5 to 10 validated individuals were pooled to construct sequencing libraries with an insert size of 350 bp. These libraries were sequenced on the Illumina platform (PE150) by Novogene Co., Ltd. (Beijing, China), ensuring a minimum data output of 2 Gbp per sample.

### 2.3. Mitochondrial Genome Assembly and Annotation

De novo assembly of mitochondrial genomes was performed using NOVOPlasty v4.3.1 [18], using the obtained *COI* sequence as a seed and a K-mer value set to 33. To verify the circularity of the assembled mitogenomes, the terminal sequences were checked for overlap, and the assembly results were mapped against closely related reference genomes to detect potential assembly errors. To further assess the completeness and accuracy of the assemblies, raw sequencing reads were mapped back to the respective assembled mitochondrial genomes using BWA-MEM (v0.7.17) [19]. The coverage depth at each site was calculated using SAMtools (v1.9) [20], and coverage plots were generated using the R package ggplot2 (v4.0.3). Assembly quality was evaluated based on mean coverage depth, coverage uniformity, and the presence of zero-coverage gaps.

Gene annotation and prediction of tRNA secondary structures were carried out using the MITOS2 web server (http://mitos.bioinf.uni-leipzig.de/, accessed on 1 December 2026) on the Galaxy platform [21,22,23]. The annotation results were visualized using Geneious Prime 2019.2 software [24]. Nucleotide composition, codon usage and relative synonymous codon usage (RSCU) of protein-coding genes (PCGs) were analyzed using PhyloSuite v1.2.2 [25]. The circular map of the mitochondrial genome was illustrated using the software tools of OrganellarGenomeDRAW (https://chlorobox.mpimp-golm.mpg.de/OGDraw.html, accessed on 4 December 2026) [26]. Nucleotide composition bias was evaluated by calculating the AT-skew and GC-skew indices. For a given DNA sequence or window, the AT-skew was defined as (A − T)/(A + T), and the GC-skew was defined as (G − C)/(G + C), where A, T, G, and C denote the counts of the four nucleotides. These metrics range from −1 to +1, with positive values indicating an excess of A over T (or G over C), and negative values indicating the opposite [27].

### 2.4. Phylogenetic Analysis

Ten mitochondrial genome sequences of Isotomidae species were downloaded from the GenBank database. These were combined with three newly sequenced mitogenomes from this study, resulting in a total of 13 ingroup taxa representing Isotomidae: five Collembola species—*Salina celebensis* (Schäffer, 1898) (family Paronellidae), *Oncopodura yosiiana* (Szeptycki, 1977) (family Oncopoduridae), *Lepidocyrtus fimetarius* (Gisin, 1964) (family Entomobryidae), *Aphaenomurus interpositus* (Potapov & Stebaeva, 1997) (family Tomoceridae), and *Allacma fusca* (Linnaeus, 1758) (order Symphypleona) were selected as outgroups. These outgroups represent major lineages within Collembola that are well-established as external to Isotomidae, ensuring a robust phylogenetic framework for testing ingroup relationships [7].

The nucleotide sequences of the 13 PCGs and two rRNA genes (16S and 12S rRNA) were individually extracted using PhyloSuite v2.0.dev2. To maintain the open reading frame and ensure biological accuracy, the 13 PCGs were aligned using the “translation alignment” option, where sequences were first translated into amino acids, aligned with MAFFT v7.313 [25] and then back-translated into nucleotides. The two rRNA genes were aligned directly as nucleotide sequences. To evaluate the phylogenetic signal, substitution saturation was assessed using Xia’s test implemented in DAMBE [28], where the observed index of substitution saturation (Iss) was compared with the critical Iss (Iss.c) under both symmetrical and extremely asymmetrical tree topologies. Poorly aligned regions were removed using trimAl v1.2 [29] with the “automated1” option. Two supermatrices were concatenated: (a) the nucleotide sequences of 13 PCGs, (b) the nucleotide sequences of 13 PCGs and two rRNA genes. The dataset was divided into multiple partitions based on gene type. We utilized ModelFinder implemented in PhyloSuite to determine the optimal partitioning schemes and the best-fit evolutionary models for each partition. This approach specifically accounts for rate heterogeneity across different sites and lineages, ensuring the accuracy of the phylogenetic reconstruction. Bayesian inferences were performed in MrBayes [30] using two simultaneous Markov Chain Monte Carlo runs, with 4 chains of 10,000,000 generations each, sampling trees every 1000 generations, applying the optimal partitioning schemes and models. Posterior probabilities (BPP) were interpreted as statistical support values for the tree resulting from Bayesian inference. Maximum Likelihood analyses were performed using IQ-TREE implemented in PhyloSuite, applying the best-fit partition models determined by ModelFinder [31]. Nodal support was assessed using 1000 standard non-parametric bootstrap replicates. Phylogenetic trees were visualized and annotated using FigTree v1.4.4.

## 3. Results

### 3.1. Genome Organization and Composition

**Figure 1:**
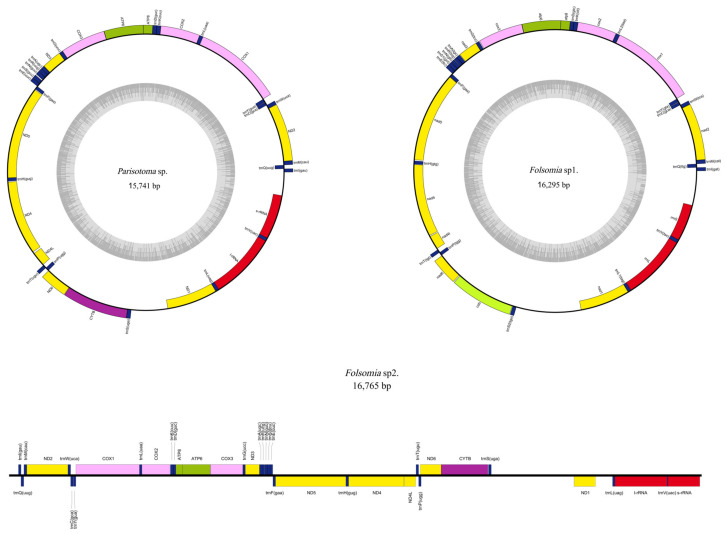
Mitochondrial genome maps of *Parisotoma* sp., *Folsomia* sp. 1, and *Folsomia* sp. 2.

The complete circular mitogenomes of *Parisotoma* sp. and *Folsomia* sp. 1 were successfully assembled, with lengths of 15,741 bp and 16,295 bp, respectively (Figure 1). The mitogenome of *Folsomia* sp. 2 was assembled as a linear contig of 16,765 bp due to the inability to resolve the typically repetitive control region. All mitochondrial genome assemblies demonstrated high and uniform sequencing coverage (Supplementary Figure S3). The assemblies showed >99.9% coverage at ≥10X depth with no coverage gaps, indicating complete and reliable assembly. All three mitogenomes contained 37 genes, including 13 protein-coding genes, 22 transfer RNA (*tRNA*) genes, and 2 ribosomal RNA (*rRNA*) genes. All tRNAs were able to form the typical cloverleaf secondary structure. The tRNA gene order in these three mitogenomes was identical to that of other previously published Isotomidae species, further confirming the structural conservation of the mitogenome within this family. Comparative analysis of the 13 Isotomidae mitogenomes revealed consistently high A + T content (63.7–77.3%) and negative GC-skew values across all species (Table 1).

### 3.2. Protein-Coding Genes and Codon Usage Frequency

A further comparative analysis of codon usage within the protein-coding genes across all Isotomidae mitogenomes was conducted. The results revealed that there are seven types of start codons (ATG, ATT, ATA, GTG, ATC, TTG, TTA) and two types of complete stop codons (TAA, TAG), as well as two types of incomplete stop codons (TA, T) in the Isotomidae mitogenomes. All the 13 Isotomidae mitogenomes exhibited almost identical trends in the usage frequency of the 13 protein-coding genes, with *Cys* showing the lowest usage frequency while *Ile* or *Phe* showing the highest usage frequency (Table S6).

### 3.3. Phylogenetic Analysis of the Family Isotomidae

**Figure 2:**
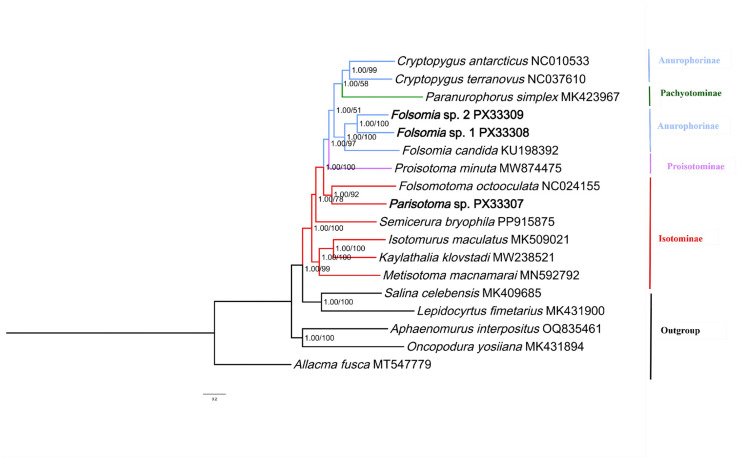
Phylogenetic tree of Isotomidae inferred from the concatenated dataset of 13 PCGs and 2 rRNAs using both Bayesian Inference (BI) and Maximum Likelihood (ML) methods. Support values on nodes indicate Bayesian posterior probabilities (BPP) and maximum likelihood bootstrap support (BS), respectively. The newly sequenced mitogenomes are highlighted in bold.

Phylogenetic relationships were reconstructed using both Bayesian Inference (BI) and Maximum Likelihood (ML) based on the concatenated PCG + RNA dataset representing 18 species. The topologies recovered by both methods were identical, showing broad congruence across major lineages. Analyses based on the PCG-only dataset also yielded a congruent topology, supporting the stability of these results (Supplementary Figure S2). Figure 2 presents the topology inferred from the BI analysis, with nodal support values indicated as Bayesian Posterior Probabilities (BPP) followed by ML Bootstrap Support (BS). The family Isotomidae was recovered as a strongly supported monophyletic group (1.00/99). Basally, the family split into two deep lineages: the “Core Isotominae” clade, comprising *Isotomurus maculatus*, *Metisotoma macnamarai*, and *Kaylathalia klovstadi*, which received maximal support in both analyses (1.00/100), and a second large clade comprising the non-core Isotominae genera alongside the remaining subfamilies within the Isotomidae family, which was also robustly supported (1.00/99).

Within this second major lineage, the subfamily Isotominae was revealed as paraphyletic within the family Isotomidae, as the genera *Semicerura*, *Parisotoma*, and *Folsomotoma* did not cluster with the type genus *Isotomurus*. Specifically, *S. bryophila* was recovered as the basal-most lineage of this clade (1.00/78), followed by a highly supported subclade formed by *Parisotoma* sp. and *F. octooculata* (1.00/92). Regarding the derived subfamilies, the analyses revealed a complex evolutionary history characterized by the independent position of Proisotominae and the paraphyly of Anurophorinae. The phylogenetic analyses also evidenced that *P. minuta* likely diverges as an independent sister group to the combined Anurophorinae + Pachyotominae clade (1.00/97), although further sampling is necessary to confirm the distinctness of this subfamily.

The phylogenetic position of *P. simplex* (currently classified in Pachyotominae) suggests that this species may be misclassified and potentially belongs to Anurophorinae, as it was recovered as the sister group to *Cryptopygus* (Anurophorinae) and nested within a clade including the genus *Folsomia*. Our phylogenetic analyses showed that *P. simplex* (currently classified in Pachyotominae) was nested into Anurophorinae with high supports by BI analysis (BPP = 1.00) but notably low support by ML bootstrap values (BS = 58 and 51, respectively). This discrepancy, combined with the low ML support, suggests that the phylogenetic signal for the placement of *P. simplex* within the mitochondrial dataset remains inconclusive, possibly reflecting a history of rapid radiation or limited signal in the mitogenomic data.

## 4. Discussion

### 4.1. Structural Conservation and Novelty of the Newly Sequenced Mitogenomes

The first mitogenomes of *Parisotoma* and two new mitogenomes of *Folsomia* reported here exhibit the highly conserved architecture typical of metazoans and Collembola [42,43]. Their sizes, tRNA and PCG gene orders, and composition align with previously published Isotomidae mitogenomes, underscoring the evolutionary stability of mitogenomic organization in this group. The slightly larger size of the mitogenome of *Folsomia* sp. 1 and *Folsomia* sp. 2 may be attributed to expansions in non-coding regions, a common driver of mitogenomic size variation. The absence of any gene rearrangements in both PCGs and tRNAs further confirms the conserved nature of mitogenomes within the family Isotomidae.

### 4.2. Phylogenetic Reconstructions Challenge the Traditional Subfamily Classification

The family Isotomidae is widely accepted as comprising four subfamilies: Proisotominae, Anurophorinae, Isotominae, and Pachyotominae based on body morphology [1,44]. Unlike previous studies that sampled species from only two or three subfamilies [15,39], the phylogenetic reconstruction in the present study included 13 mitogenomes covering all four subfamilies of Isotomidae. However, as our sampling of species and previous phylogenetic studies [16] did not include the type species for each subfamily, these phylogenetic interpretations must be handled with care. While our results yield a topology largely congruent with evidence from previous molecular datasets but challenge current morphological classifications, no definitive taxonomic conclusions can be formulated at this stage. This discordance highlights the need for a major re-evaluation of subfamilial boundaries within Isotomidae [39].

The most robust finding of our study is the deep paraphyly of the subfamily Isotominae, a conclusion consistently supported by congruent topologies in both the PCG + RNA (Figure 2) and PCG-only datasets (Supplementary Figure S2). In contrast to traditional classification, three genera of Isotominae, i.e., *Parisotoma* sp., *S. bryophila*, and *F. octooculata* were recovered as closely related to the other three subfamilies rather than the core Isotominae clade, which includes the type genus *Isotomurus* alongside *Metisotoma* and *Kaylathalia*. Consistent with the recent findings of Xie et al. [15], who first highlighted the taxonomic instability of *S. bryophila*, our analysis recovered *S. bryophila* as the earliest diverging lineage of this non-core assemblage. Expanding upon this, our inclusion of *Parisotoma* sp. further revealed that *Parisotoma* sp. and *F. octooculata* form a distinct monophyletic lineage, which stands as the sister group to the clade comprising the other three subfamilies.

### 4.3. Morphological Implications and Taxonomic Re-Evaluation

The phylogenetic distinctness of *Parisotoma* from the type genus *Isotomurus* fundamentally questions the validity of the current subfamilial framework. Traditionally, *Parisotoma* was placed within Isotominae based on plesiomorphic characters such as the presence of a Postantennal Organ (PAO) and a complete furca with a mucro [14]. However, our topology suggests that these traits are symplesiomorphies retained from the common ancestor of Isotomidae, rather than synapomorphies linking *Parisotoma* sp. to *I. maculatus*. This separation is further corroborated by biogeography and chaetotaxy: while the sister genus of *Parisotoma*, i.e., *F. octooculata*, is exclusively distributed in the Southern Hemisphere [45], their robust clustering is supported by a shared tendency toward chaetotaxy reduction (paurosis), standing in sharp contrast to the densely setose condition of the core Isotominae. Consequently, the current concept of Isotominae appears excessively broad, necessitating a re-evaluation of the taxonomic status of *Parisotoma* sp. (and *F. octooculata*).

For the subfamily Proisotominae, the taxonomic placement of the genus *Proisotoma* has long been a subject of debate. Traditionally, *Proisotoma* serves as the type genus of the subfamily Proisotominae, characterized morphologically by a reduced yet functional furca and a distinct manubrium [14]. However, the boundary between Proisotominae and Anurophorinae is frequently blurred, as furcal reduction is evolutionarily labile and prone to convergence [16]. Our analysis recovered *P. minuta* as a robust independent lineage forming the sister group to the combined Anurophorinae + Pachyotominae clade. The intermediate position of *P. minuta* recovered in our study suggests that while it is genetically distinct from Anurophorinae, it shares a more recent common history with them than with Isotominae. While this topology should be considered provisional due to limited taxon sampling, our results provide preliminary molecular evidence supporting the view of Proisotominae as an evolutionary grade rather than a natural clade. However, phylogenetic sampling of additional taxa is required to confirm the stability of this relationship [39].

Furthermore, the recovery of *P. simplex* nested within Anurophorinae challenges the monophyly of Anurophorinae and provides molecular support for the hypothesis that Pachyotominae is not an ancient, independent subfamily but a specialized lineage derived from within the Anurophorinae [14,46]; however, it needs to be noted that the *Paranurophorus simplex* (currently assigned to Pachyotominae) is not the type genus of Pachyotominae, and the maximum likelihood bootstrap support values were notably low. In this context, the characteristic cuticular hardening of *P. simplex* may represent an adaptive specialization to specific microhabitats (e.g., desiccation resistance) that evolved from a *Cryptopygus*-like ancestor [47], reinforcing the biological validity of this non-traditional grouping.

Although this study increased the publicly available mitochondrial genome data of Isotomidae to 13 sequences, covering all four subfamilies of the family, the overall sampling remains relatively limited, especially for key groups such as Pachyotominae, which is represented by only a single species with reduced mitogenome length. The limited taxonomic coverage may influence the stability of phylogenetic inferences, particularly in resolving relationships within subfamilies. Despite these limitations, our study provides important mitogenomic data and phylogenetic insights that challenge the current subfamilial classification of Isotomidae and calls for future studies integrating nuclear loci, mitogenomic data, and morphological evidence.

## 5. Conclusions

This study reports the first complete mitogenome for the genus *Parisotoma* and expands the available genomic resources for the genus *Folsomia* by providing two newly sequenced mitogenomes. While comparative genomic analyses confirmed high structural conservation within the family, our phylogenetic reconstruction—representing all four subfamilies—reveals fundamental conflicts with the traditional morphology-based classification. Specifically, Isotominae is recovered as deeply paraphyletic: *Parisotoma* and allied genera form a distinct lineage closer to the derived subfamilies (Anurophorinae and Proisotominae) than to the type genus *Isotomurus*, suggesting that shared morphological traits like the furca are likely symplesiomorphies. Furthermore, the recovery of *P. minuta* as an independent sister lineage to the Anurophorinae + Pachyotominae clade, combined with the nesting of Pachyotominae within Anurophorinae, highlights the instability of current subfamilial definitions and the need for taxonomic re-evaluation of specific genera. These results underscore the critical need for an integrative taxonomic revision of Isotomidae that combines dense molecular sampling with careful morphological re-assessment. Future work should focus on generating genome-scale data for key taxa, particularly within the Proisotominae and Pachyotominae lineages, and on developing a new, phylogenetically informed classification system for the family. This work provides a foundational framework and essential genomic resources for such future studies.

## Figures and Tables

**Table 1:** Mitogenome characteristics and base composition of taxa used in this study.

Family	Subfamily	Species	Base Composition					AT Skew	GC Skew	Size (bp)	Accession No.	Reference
			T%	C%	A%	G%	AT%					
Isotomidae	Isotominae	*Metisotoma macnamarai* (Folsom, 1918)	33.3	17.4	37.1	11.8	70.4	0.054	−0.192	15,177	MN592792	[32]
Isotomidae	Isotominae	*Isotomurus maculatus* (Schäffer, 1896)	29.2	21.3	34.4	15.1	63.6	0.082	−0.170	15,263	MK509021	[33]
Isotomidae	Isotominae	*Semicerura bryophila* (Cassagnau, 1959)	36.6	15.0	38.0	10.2	74.6	0.019	−0.190	15,247	PP915875	[15]
Isotomidae	Isotominae	*Folsomotoma octooculata* (Willem, 1901)	35.6	14.6	38.9	10.9	74.5	0.044	−0.145	15,338	NC024155	[34]
Isotomidae	Isotominae	*Kaylathalia klovstadi* (Carpenter, 1902)	31.6	18.8	36.1	13.5	67.7	0.066	−0.164	15,485	MW238521	[35]
Isotomidae	Isotominae	*Parisotoma* sp.	32.3	18.8	37.9	11.0	70.2	0.080	−0.262	15,741	PX733307	This study
Isotomidae	Anurophorinae	*Folsomia* sp. 1	32.7	17.9	36.4	13.0	69.1	0.054	−0.159	16,295	PX733308	This study
Isotomidae	Anurophorinae	*Folsomia* sp. 2	34.2	15.8	38.6	11.5	72.8	0.060	−0.158	16,765	PX733309	This study
Isotomidae	Anurophorinae	*Cryptopygus antarcticus* (Willem, 1901)	33.4	16.7	37.5	12.4	70.9	0.058	−0.148	15,297	NC010533	[36]
Isotomidae	Anurophorinae	*Cryptopygus terranovus* (Wise, 1967)	35.8	15.9	36.7	11.7	72.5	0.012	−0.152	15,352	NC037610	[37]
Isotomidae	Anurophorinae	*Folsomia candida* (Willem, 1902)	32.9	17.3	37.3	12.5	70.2	0.063	−0.161	15,147	KU198392	[38]
Isotomidae	Proisotominae	*Proisotoma minuta* (Tullberg, 1871)	28.5	21.3	35.9	13.7	64.4	0.115	−0.217	15,930	MW874475	[39]
Isotomidae	Pachyotominae	*Paranurophorus simplex* (Denis, 1929)	35.4	14.9	39.3	10.5	74.7	0.052	−0.173	9,518	MK423967	[7]
Oncopoduridae	——	*Oncopodura yosiiana* (Szeptycki, 1977)	35.3	13.7	41.1	10.0	76.4	0.076	−0.156	14,808	MK431894	[7]
Entomobryidae	Lepidocyrtinae	*Lepidocyrtus fimetarius* (Gisin, 1964)	30.8	19.4	38.0	11.7	68.8	0.105	−0.248	14,698	MK431900	[7]
Paronellidae	Salininae	*Salina celebensis* (Schäffer, 1898)	33.0	17.6	36.7	12.6	69.7	0.053	−0.166	14,788	MK409685	[7]
Sminthuridae	——	*Allacma fusca* (Linnaeus, 1758)	32.6	16.4	39.3	11.6	71.9	0.093	−0.171	15,111	MT547779	[40]
Tomoceroidae	——	*Aphaenomurus interpositus* (Potapov & Stebaeva, 1997)	34.5	15.4	39.1	11.0	73.6	0.063	−0.167	15,090	OQ835461	[41]

## Data Availability

The genome sequence data that support the findings of this study have been submitted to GenBank of NCBI and obtained accession numbers (PX733307, PX733308, PX733309). The raw sequencing data have been deposited in the NCBI Sequence Read Archive (SRA) database under BioProject accession number PRJNA1416235.

## Supplementary Materials

The following supporting information can be downloaded at: https://www.mdpi.com/article/10.3390/genes17020166/s1, Figure S1: High-resolution images of the voucher specimens (*Parisotoma* sp., *Folsomia* sp. 1, and *Folsomia* sp. 2) used in this study; Figure S2: BI phylogenetic tree of Isotomidae based on concatenated nucleotide sequences of 13 PCGs; Figure S3: Per-base sequencing depth across the mitochondrial genomes of (A) *Parisotoma* sp., (B) *Folsomia* sp. 1, and (C) *Folsomia* sp. 2. The plot confirms the high quality of the de novo assembly and supports the genetic consistency of the pooled specimens; Table S1: List of primers used for PCR amplification; Tables S2–S4: Summary of genetic components of *Parisotoma* sp., *Folsomia* sp. 1, and *Folsomia* sp. 2; Table S5: Details of individuals used for pooled mitogenome sequencing and their *COI* sequence identity; Table S6: Amino acid usage of mitochondrial protein-coding genes for the species used in this study.
