# Supplementary material for: Phylogenetic Reconstructions Based on Mitogenomes Reveal the Paraphyly of the Subfamily Isotominae of Isotomidae (Collembola: Entomobryomorpha)
Source: Genes (Basel). 2026 Jan 30;17(2):166. doi: 10.3390/genes17020166 (PMC12940504; doi:10.3390/genes17020166)
Supplement: Supplementary file 1 [file genes-17-00166-s001.zip › genes-4128440-supplementary.pdf]

Supplementary Materials

Phylogenetic Reconstructions Based on Mitogenomes Reveal the Paraphyly of the Subfamily Isotominae of Isotomidae (Collembola: Entomobryomorpha)

Authors: Yuhang Cheng [1,2], Chunyu Zhang [3], Donghui Wu [1,2,4,5,*], Zhijing Xie [4,5] and Bing Zhang [3,*]

[1] Key Laboratory of Wetland Ecology and Environment, State Key Laboratory of Black Soils Conservation and Utilization, Northeast Institute of Geography and Agroecology, Chinese Academy of Sciences, Changchun 130102, China

[2] University of Chinese Academy of Sciences, Beijing 100049, China

[3] School of Grassland Science, Beijing Forestry University, Beijing 100083, China

[4] Key Laboratory of Vegetation Ecology, Ministry of Education, Northeast Normal University, Changchun 130024, China

[5] State Environmental Protection Key Laboratory of Wetland Ecology and Vegetation Restoration, School of Environment, Northeast Normal University, Changchun 130024, China

* Correspondence: wudonghui@iga.ac.cn (D.W.); bzhang3@bjfu.edu.cn (B.Z.)

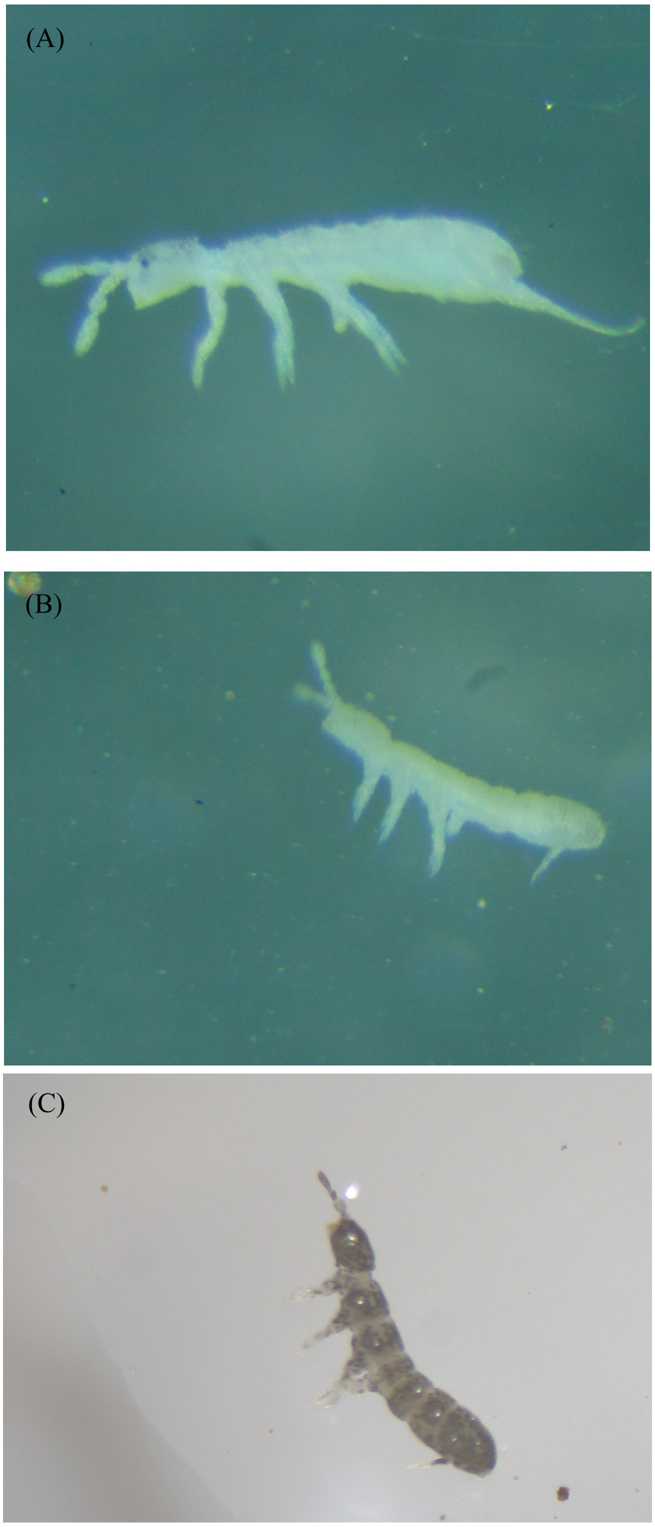

Figure S1. High-resolution images of the voucher specimens used in this study. (A) *Parisotoma* sp. (Specimen No.: SHB-2023-FS01); (B) *Folsomia* sp. 1 (Specimen No.: SHB-2023-FS02); (C) *Folsomia* sp. 2 (Specimen No.: SHB-2023-FS03). Collection site: Saihanba Mechanical Forest Farm, Chengde City, Hebei Province, China; Collection date: July 2023.

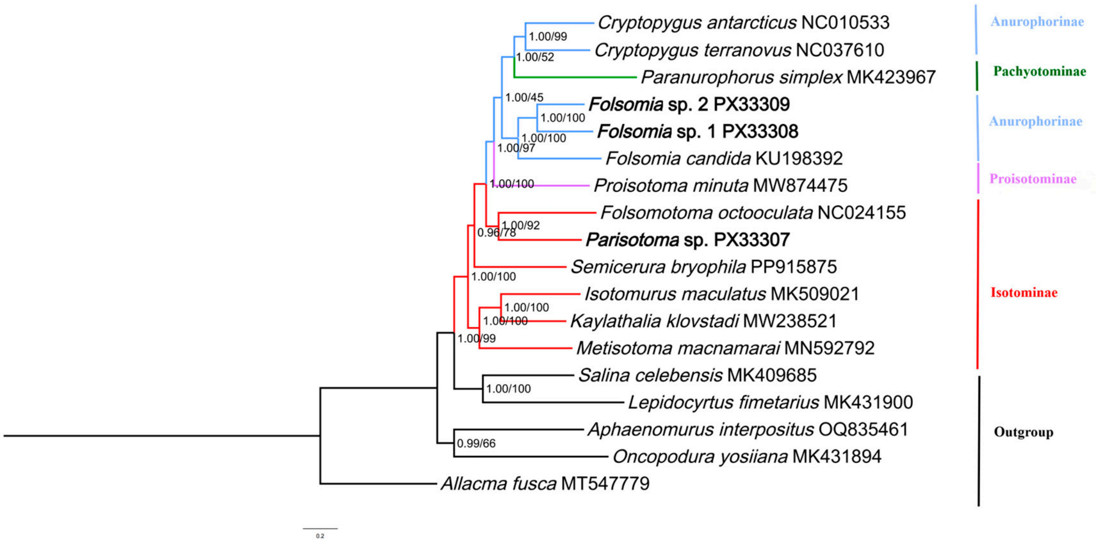

Figure S2. Phylogenetic tree of Isotomidae inferred from the concatenated dataset of 13 PCGs using both Bayesian Inference (BI) and Maximum Likelihood (ML) methods. Support values on nodes indicate Bayesian posterior probabilities (BPP) and maximum likelihood bootstrap support (BS), respectively. The newly sequenced mitogenomes are highlighted in bold.

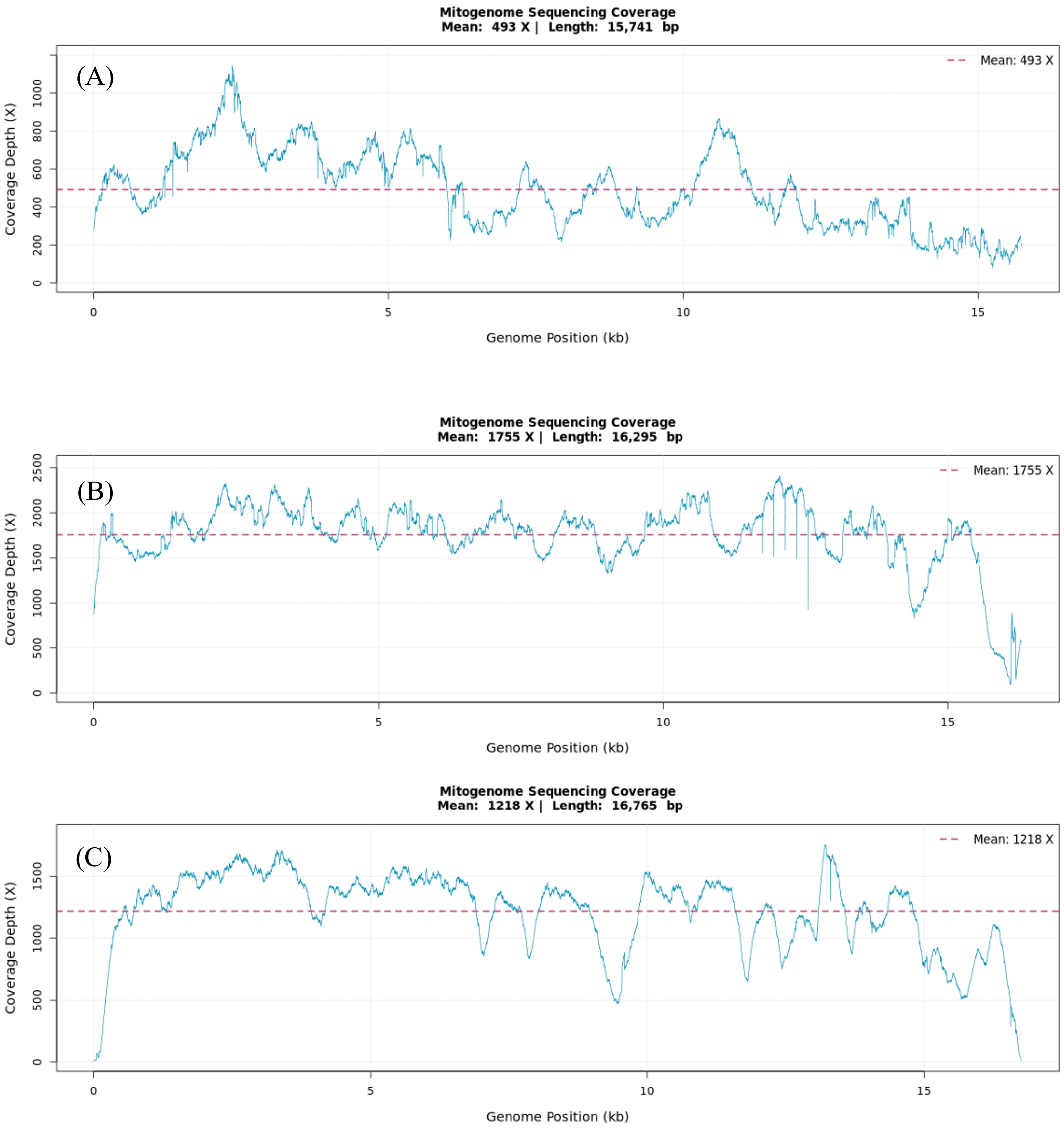

Figure S3. Per-base sequencing depth across the mitochondrial genomes (A) *Parisotoma* sp. (B) *Folsomia* sp. 1 (C) *Folsomia* sp. 2 The x-axis represents the genomic position in base pairs (bp), and the y-axis represents the sequencing depth per base. The horizontal dashed lines represent the mean sequencing depth for each assembled mitogenome. The absence of significant depth fluctuations or zero-coverage gaps confirms the high quality of the de novo assembly and supports the genetic consistency of the pooled specimens used for sequencing.

Table S1. List of primers used for PCR amplification

**Primer Name**	**Sequence (5'–3')**	**Gene**
LCO1490	GGTCAACAAATCATAAAGATATTGG	COI
HCO2198	TAAACTTCAGGGTGACCAAAAAATCA	COI

Table S2. Summary of genetic components of *Parisotoma* sp.

Gene	Position		Size	Codon		Strand
	From	To		Start	Stop	
trnI	1	63	63			H
trnQ	62	130	69			L
trnM	130	198	69			H
nad2	199	1200	1002	ATG	TAA	H
trnW	1202	1269	68			H
trnC	1269	1331	63			L
trnY	1334	1398	65			L
cox1	1398	2931	1534	TTG	T	H
trnL2	2932	2994	63			H
cox2	2995	3677	683	ATA	TA	H
trnK	3678	3747	70			H
trnD	3747	3815	69			H
atp8	3816	3980	165	ATC	TAA	H
atp6	3980	4657	678	ATA	TAA	H
cox3	4657	5445	789	ATG	TAA	H
trnG	5445	5507	63			H
nad3	5505	5852	348	ATA	TAG	H
trnA	5851	5911	61			H
trnR	5912	5974	63			H
trnN	5975	6041	67			H
trnS1	6058	6128	71			H
trnE	6129	6191	63			H
trnF	6191	6254	64			L
nad5	6265	7974	1710	ATT	TAA	L
trnH	7972	8035	64			L
nad4	8049	9413	1365	ATG	TAA	L
nad4L	9444	9707	264	ATT	TAA	L
trnT	9719	9783	65			H
trnP	9784	9850	67			L
nad6	9853	10335	483	ATA	TAA	H
cytb	10339	11475	1137	ATG	TAA	H
trnS2	11474	11543	70			H
nad1	12206	13138	933	ATA	TAA	L
trnL1	13139	13202	64			L
rrnL	13154	14476	1323			L
trnV	14433	14496	64			L
rrnS	14493	15317	825			L

Table S3. Summary of genetic components of *Folsomia* sp. 1

Gene	Position		Size	Codon		Strand
	From	To		Start	Stop	
trnI	1	64	64			H
trnQ	63	130	68			L
trnM	129	196	68			H
nad2	197	1193	997	ATG	T	H
trnW	1194	1259	66			H
trnC	1259	1320	62			L
trnY	1324	1390	67			L
cox1	1393	2926	1534	ATG	T	H
trnL2	2927	2990	64			H
cox2	2991	3678	688	ATT	T	H
trnK	3679	3749	71			H
trnD	3749	3812	64			H
atp8	3813	3977	165	ATC	TAA	H
atp6	3974	4654	681	ATA	TAA	H
cox3	4654	5442	789	ATG	TAA	H
trnG	5451	5512	62			H
nad3	5513	5854	342	ATA	TAA	H
trnA	5860	5923	64			H
trnR	5923	5983	61			H
trnN	5986	6049	64			H
trnS1	6050	6116	67			H
trnE	6117	6181	65			H
trnF	6189	6254	66			L
nad5	6255	7941	1687	ATT	T	L
trnH	7960	8024	65			L
nad4	8030	9379	1350	ATG	TAG	L
nad4L	9396	9656	261	ATA	TAA	L
trnT	9678	9739	62			H
trnP	9740	9803	64			L
nad6	9812	10291	480	ATT	TAA	H
cytb	10309	11424	1116	ATA	TAA	H
trnS2	11431	11500	70			H
nad1	12699	13628	930	GTG	TAA	L
trnL1	13629	13691	63			L
rrnL	13669	14942	1274			L
trnV	14908	14973	66			L
rrnS	14971	15658	688			L

Table S4. Summary of genetic components of *Folsomia* sp. 2

Gene	Position		Size	Codon		Strand
	From	To		Start	Stop	
trnI	225	288	64			H
trnQ	287	354	68			L
trnM	359	426	68			H
nad2	427	1425	999	ATG	TAA	H
trnW	1424	1489	66			H
trnC	1489	1549	61			L
trnY	1554	1616	63			L
cox1	1618	3156	1539	ATG	TAA	H
trnL2	3152	3212	61			H
cox2	3213	3896	684	ATT	TAG	H
trnK	3903	3972	70			H
trnD	3973	4035	63			H
atp8	4036	4197	162	ATT	TAA	H
atp6	4191	4871	681	ATG	TAA	H
cox3	4871	5658	788	ATG	TA	H
trnG	5658	5719	62			H
nad3	5717	6061	345	ATA	TAG	H
trnA	6060	6124	65			H
trnR	6124	6184	61			H
trnN	6187	6250	64			H
trnS1	6251	6317	67			H
trnE	6318	6382	65			H
trnF	6386	6448	63			L
nad5	6448	8153	1706	GTG	TA	L
trnH	8154	8216	63			L
nad4	8217	9560	1344	ATA	TAA	L
nad4L	9567	9842	276	ATT	TAA	L
trnT	9851	9913	63			H
trnP	9914	9975	62			L
nad6	9954	10460	507	ATT	TAA	H
cytb	10460	11593	1134	ATG	TAA	H
trnS2	11604	11673	70			H
nad1	13678	14193	516	ATA	TAA	L
trnL1	14608	14668	61			L
rrnL	14622	15932	1311			L
trnV	15888	15953	66			L
rrnS	15950	16720	771			L

Table S5. Details of individuals used for pooled mitogenome sequencing and their COI sequence identity.

**Species**	**Number of Individuals**	**Sequence Identity (%)**	**Morphological Consistency**	**Accession No.**
Parisotoma sp.	4	100%	Confirmed	PX733307
Folsomia sp. 1	4	100%	Confirmed	PX733308
Folsomia sp. 2	4	100%	Confirmed	PX733309

Table S6. Amino acid usage of mitochondrial protein-coding genes for the species used in this study

**AA**	***Folsomia* sp. 2**	***Paranurophorus simplex***	***Folsomia* sp. 1**	***Parisotoma* sp.**	***Proisotoma minuta***	***Folsomia candida***	***Cryptopygus terranovus***	***Cryptopygus antarcticus***	***Kaylathalia klovstadi***	***Folsomotoma octooculata***	***Semicerura bryophila***	***Isotomurus maculatus***	***Metisotoma macnamarai***	***Oncopodura yosiiana***	***Lepidocyrtus fimetarius***	***Salina celebensis***	***Allacma fusca***	***Aphaenomurus***
Ala	197	117	224	204	238	200	207	203	231	170	177	247	206	137	186	199	163	145
Cys	30	21	35	28	26	28	38	32	36	33	28	43	33	31	32	39	38	26
Asp	64	51	62	63	61	67	69	66	66	63	61	62	72	66	68	65	60	63
Glu	81	63	80	85	80	83	78	80	77	83	81	81	71	77	77	80	76	80
Phe	351	272	345	335	321	378	353	353	345	346	386	326	331	415	372	364	355	377
Gly	223	167	248	240	265	231	224	230	253	224	220	250	232	206	240	240	209	203
His	68	59	69	71	71	77	70	70	83	71	72	78	76	70	77	74	68	68
Ile	340	314	313	357	289	323	360	328	294	363	363	278	359	393	329	322	331	368
Lys	106	86	96	95	91	110	92	97	88	101	104	90	96	127	115	101	118	126
Leu2	297	322	286	327	301	315	348	327	303	387	372	255	330	401	298	325	290	372
Leu1	195	84	238	194	251	209	192	192	239	122	152	302	192	142	243	220	248	167
Met	245	189	235	265	215	221	228	256	209	318	250	196	236	269	232	204	262	248
Asn	155	134	148	160	132	149	161	164	131	179	175	131	150	184	158	150	154	179
Pro	125	94	135	137	146	133	132	137	132	121	125	140	137	116	140	133	130	128
Gln	63	47	73	65	67	64	69	65	74	64	63	72	71	69	67	71	65	66
Arg	51	38	56	58	60	54	55	56	59	55	55	58	55	52	54	55	50	49
Ser2	222	183	218	200	216	225	225	225	214	211	238	206	240	235	226	228	248	240
Ser1	94	85	109	103	129	112	123	125	128	108	123	130	118	118	107	111	134	132
Thr	190	154	210	210	221	216	189	193	203	187	168	215	194	161	202	199	180	187
Val	217	124	232	237	257	223	211	233	270	226	205	280	211	177	215	261	238	201
Trp	89	65	90	90	91	92	94	90	95	92	90	92	92	90	84	90	89	87
Tyr	145	120	161	161	149	136	154	163	158	154	155	157	156	144	156	147	154	156
